# Assessment of sensorimotor and strength related function of breast cancer patients during systemic drug therapy: a prospective observational study

**DOI:** 10.1186/s12885-023-11494-x

**Published:** 2023-10-14

**Authors:** Alper Tuğral, Zeynep Arıbaş, Murat Akyol, Yeşim Bakar

**Affiliations:** 1 Department of Physiotherapy and Rehabilitation, Faculty of Health Sciences, Izmir Bakırçay University, Izmir, Turkey; 2 Department of Medical Oncology, Faculty of Medicine, Izmir Bakırçay University, Izmir, Turkey

**Keywords:** Breast cancer, Chemotherapy, Chemotherapy induced peripheral neuropathy, Fatigue, Handgrip strength, Peripheral muscle strength, Semmes–weinstein monofilament test, Minnesota manual dexterity test

## Abstract

**Background:**

Chemotherapy is a well-known risk factor for sensorial and motor disturbances. Chemotherapy induced peripheral neuropathy (CIPN) which predominantly affects sensory nerves might cause a diminished fine motor function. This prospective observational study aimed to assess the sensorimotor functions of breast cancer patients before, during, and after chemotherapy.

**Methods:**

A total of 56 breast cancer patients were evaluated at three different times as follows: T1 (before chemotherapy), T2 (middle chemotherapy), and T3 (completion of chemotherapy). Motor function was assessed with handgrip strength (HGS), peripheral muscle strength (PMS), and the Minnesota Manual Dexterity Test (MMDT). Semmes Weinstein Monofilament Test (SWMT) was performed to assess the sensory function. Fatigue was evaluated with the European Organization for Research and Treatment of Cancer Quality of Life Module Cancer Related Fatigue (EORTC-QLQ-FA12), respectively.

**Results:**

HGS and MMDT were found significant (χ^2^: 11.279, *p* = 0.004 and χ^2^: 9.893, *p* = 0.007, respectively) whereas PMS was not found significant (F (2,110) = 1.914, *p* = 0.152). Pairwise comparisons with Bonferroni adjustments revealed that HGS was found significant between T1 and T3, while significant results were obtained between T1 and T2 as well as T2 and T3 in MMDT (*p* = 0.01 and *p* = 0.042). There were significant results in some reference points of SWMT, though they were not found after pairwise comparisons with Bonferroni adjustment (*p* > 0.05). Fatigue was found significantly increased from T1 through T3 (Median: 19.44 vs 27.77, z: -2.347, *p* = 0.019, Wilcoxon test).

**Conclusion:**

Our study showed that decreased handgrip strength and fine motor function, as well as increased fatigue, are evident during the chemotherapy. SWMT can be an optional assessment in the context of tracking changes in cutaneous sensation during chemotherapy due to its non-invasive, cheap, and easily repeatable features among cancer patients. To preserve functional capacity as well as independence in daily living, precautions and follow up assessments during the systemic therapy process should be integrated as early as possible to prevent future deteriorations in daily life for patients who undergo chemotherapy.

**Trial registration:**

NCT04799080.

## Introduction

Chemotherapy is a typical and effective treatment method for cancer patients due to its proven efficacy in survival. Yet, some chemotherapy agents such as taxanes, frequently used in breast cancer, and platins might have extremely harmful neurotoxic adverse effects that can harm neuronal structures via glial damage, inflammation, mitochondrial dysfunction, and other mechanisms [1, 2]. Chemotherapy-induced peripheral neuropathy (CIPN), which usually emerges as sensory impairments and ultimately reduces functional ability and quality of life in cancer survivors, is known as a side effect of the specific chemotherapy agents [3]. The potential mechanism of Taxanes on CIPN was reported to be characterized by failure to achieve anaphase due to the stabilizing effect of these drugs on tubulin proteins. [4]. According to studies, 33% of individuals experience CIPN during chemotherapy, which could last for up to two years [5, 6]. Sensory neuropathy was reported to be the most prominent symptom and can have drastic impacts on activities of daily living [7, 8]. Due to impaired fine and gross motor skills, a vicious cycle of symptom aggravation may be apparent [9–12]. Decreased physical independence as well as increased perception of fatigue due to the greater energy expenditure during daily function were also reported because of CIPN among cancer patients [13].

Patients who underwent chemotherapy report that there is insufficient knowledge regarding CIPN [14]. Not only patients, but also healthcare professionals who work in oncology report the importance of detecting, managing, and monitoring CIPN, however, there is a lack of knowledge about the available assessment methods [15]. In addition, there is generally not much time to be informed about the management of CIPN in busy oncology clinical settings, and therefore patients might suffer from how to cope with mild to moderate symptoms [3]. There has not been a standardized approach for preventing CIPN, thus careful monitoring and direction are required [13].

Since CIPN was reported to cause high healthcare costs of over seventeen thousand dollars for each patient, awareness should be improved as early as possible among cancer patients undergoing chemotherapy [16]. Physical therapists can do proactive assessment and screening for both sensory and motor functions, including fine motor skills, with the right tools and knowledge [17]. In a recently published study, the key role of physical therapists was also highlighted in the management of CIPN [3]. Despite the fact that there are plenty of CIPN evaluation methods reported in the literature, there is also an obvious inconsistency that exists between the diagnosis and rating of CIPN. [18]. Though the assessment of CIPN mostly relies on valid and reliable patient-reported outcomes, it was also stated that a combination of objective and subjective items might cause a variable interpretation and might fail to objectively picture the clinical outcomes [19]. On the other hand, some tests such as electroneuromyography need specific and trained personnel as well and it is costly and time-consuming [7]. In a systematic review, it was stated that CIPN prevalence can vary in a wide range from 12.1% to 96.2% depending on assessment time and drugs [20]. Additionally, it was mentioned that the development of new functional evaluation techniques is essential for improving the definition of possible neurotoxicity. [19].

Since there is a need for longitudinal studies to understand and manage better of CIPN [21], we aimed to assess the motor, sensory, and strength-related functions of breast cancer patients undergoing chemotherapy with cost-effective and valid measures. It was also seen that measures of CIPN were mostly performed on a cross-sectional design [7]. To the best of our knowledge, there is a lack of an evaluation of fine motor function in patients at risk for CIPN undergoing chemotherapy. As previously stated, possible CIPN requires ongoing observation due to its chronic character. Therefore, the purpose of this study was to prospectively evaluate potential motor and sensory abnormalities associated with CIPN in breast cancer patients receiving chemotherapy. According to our hypothesis, patients undergoing chemotherapy would show worse fine and strength-related function than they had at baseline.

## Methods

### Study design

This study was designed as a prospective observational study and followed The Strengthening the Reporting of Observational Studies in Epidemiology (STROBE) guideline [22]. This study was completed between March 2021 and May 2022. The non-probability sampling method was used according to the inclusion criteria. All procedures and measurements in this study were performed according to the 1964 Helsinki Declaration and its later amendments or comparable ethical standards. Ethical approval was granted by Bakircay University Ethical Board of Clinical Studies with the 208/190 protocol number before the enrollment of patients.

### Patients

Patients who applied to the medical oncology unit for systemic chemotherapy were screened according to the predefined inclusion and exclusion criteria. Patients who volunteered to participate in this study were initially informed about the study process and signed consent was taken for each of them. A simple assessment form was used to collect data on demographic and clinical characteristics. The inclusion criteria were specified as being a volunteer to participate, having been diagnosed with breast cancer, and being a potential chemotherapy candidate. Exclusion criteria were set as having metastasis and/or having sensory loss caused by diabetic polyneuropathy and having illnesses or disabilities such as multiple sclerosis that include neural functional abnormalities. The mean exposure of chemotherapy drugs was calculated for each patient according to their drug regimen. Chemotherapy dose calculation was based on Body surface area (BSA) x universal doses (4 cycles of Anthracycline were applied 14 days apart 60 mg/m^2^ IV, 12 cycles of Paclitaxel were applied 7 days apart 80 mg/m^2^ IV, 4 cycles of Docetaxel were applied 21 days apart 75 mg/m^2^ IV). BSA was calculated according to the DuBois formula: BSA [m2] = Weight [kg]^0.425 × height (cm)^0.725 × 0.007184].

Eligible patients according to the aforementioned criteria were screened to enroll in this study. Although 60 patients met the requirements for inclusion, this study was completed with 56 breast cancer patients in total. One patient moved to a different place, another did not complete all evaluations, another had Sjogren's syndrome, and a fourth was lost to follow-up.

### Assessment

Repeated measurements were taken at the following three time points: Time 1 (T1): before chemotherapy, Time 2 (T2): middle chemotherapy time point (approx.8 weeks later), and Time 3 (T3): post-chemotherapy time point (12–20 weeks later after initial assessment). The main outcome measures were set as handgrip strength, peripheral muscle strength (PMS), and the Minnesota Manual Dexterity Test (MMDT) for motor function. Semmes–Weinstein Monofilament Test (SWMT) was performed for sensory evaluation. Secondarily, patients' fatigue was assessed with The European Organization for Research and Treatment of Cancer Quality of Life Module Cancer Related Fatigue (EORTC-QLQ-FA12) questionnaire in T1 and T3.

### Handgrip Strength (HGS)

HGS was assessed with a Lafayette Professional Hand Dynamometer (Model 5030L1, LaFayette Instruments, NY, USA). The standard position which can be achieved by 90-degree elbow flexion, shoulder adducted, and hand positioned in mid-prone was used to assess hand-grip strength. The patient was asked to squeeze the dynamometer as much as the patient could without holding their breath and preserving an erect posture. For each side, an average of three measurements was taken. Each measurement was separated by a 60-s delay [17]. HGS was analyzed by the mean value of the right and left sides according to the literature [23].

### Peripheral Muscle Strength (PMS)

To evaluate the PMS, quadriceps femoris (QF) muscle strength was evaluated. Patients were instructed to sit up straight with their hands crossed over their shoulders. One of the authors (Z.A.) performed the manual muscle strength test bilaterally for knee extension. 3 s were arranged to take muscle strength and peak force was recorded as Newton (N) (Lafayette Manual Muscle Tester, Lafayette, USA). Triplicate measurements were performed for each side and an average of three measurements was recorded. During the test, patients were required to preserve their erect posture without holding their breath. The 60-s interval was integrated between each measurement. PMS was also analyzed by the mean value of the right and left sides.

### Cutaneous sensation assessment

**Fig. 1:**
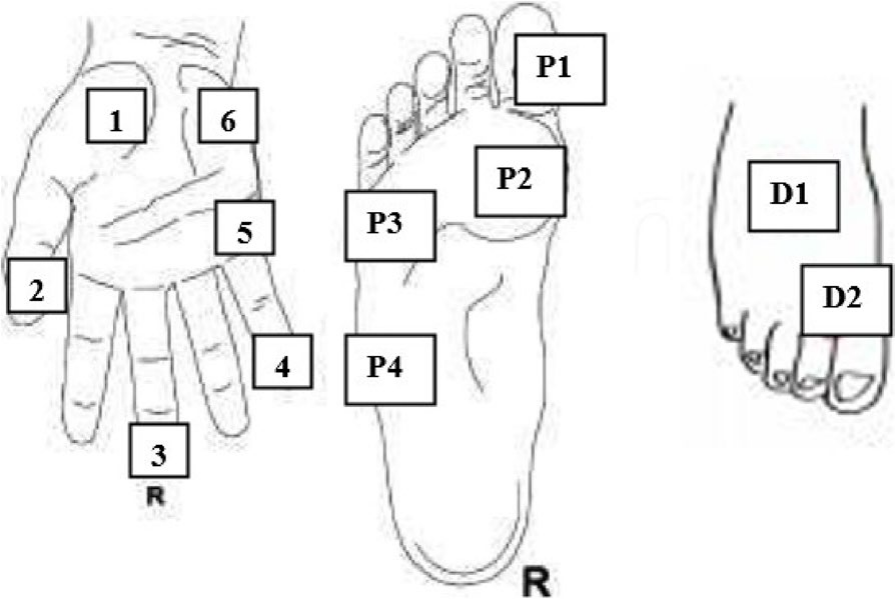
Illustration of measurement points of Semmens Weinstein Monofilament Test (SWMT). Measurements were performed both hands and feet. For hand reference points, 1,2 and 3 correspond to the palmar cutaneous branch of the Ulnar nerve for hand, while reference points 4,5 and 6 correspond to the palmar cutaneous branch of Median nerve. For foot reference points, P1 and P2 correspond to the Medial plantar nerve, P3 corresponds to the Lateral plantar nerve, P4 corresponds to the Sural nerve, D1 and D2 correspond to the Deep Fibular nerve and Dorsal lateral cutaneous nerve, respectively

The cutaneous sensation of patients was assessed with the Semmens-Weinstein Monofilament Test (SWMT) on the bilaterally palmar and plantar surface of the hand, foot, and fingers in predefined reference points (Fig. 1). Patients were informed prior to testing as they must close their eyes during testing and if anything felt, it is requested to say “yes” or “I felt”. The test was started with the smallest 2.83 monofilaments (0.07 g) and three repetitions were done. Monofilaments were applied perpendicular to the site for 1.5–2 s and then removed. If one of them was felt by the patient, the cutaneous sensation was recorded as a mechanical detection threshold [24]. The corresponding target “gram” force was recorded for each measurement point and taken into account in analyses. In addition, thresholds were used to categorize patients as having “Normal” (0.008–0.4 gr) or “Diminished protective sensation” (0.6–2 gr for hand and dorsal foot, 4–8 gr for plantar surface) according to the manufacturer’s guideline and analyzed further (Baseline® Evaluation Instruments, USA).

### Fine motor skill assessment

Minnesota Manual Dexterity Test (MMDT) (Lafayette Instruments, Lafayette, USA) has been used as a time-dependent test for different hand disabilities or injuries as well as to evaluate rehabilitation results of the hand. In essence, 60 circular plastic discs must be added to or subtracted from a board that is arranged in a 4 × 15 layout [25, 26]. One is instructed to take a standing position in front of the board. An oral command as "start" was performed and the chronometer was started. One should take hold of the disc with their right hand at the top right corner, which is the starting point, then turn the disc over and take hold of the opposite side with their left hand. When reaches the left side of the board, the pattern is altered by gripping the disc with the left hand and then switching to the right hand. When completed all discs, the chronometer is stopped, and time is recorded as “seconds”. We picked this "lifting and turning" test, which demands bilateral performance with both hands because CIPN can affect both hands.

### Fatigue assessment

The EORTC-QLQ-FA12 was used to assess fatigue in the pre-chemotherapy and post-chemotherapy periods (T1 and T3). It assesses the physical, emotional, and cognitive aspects of cancer-related fatigue. It consists of 12 items, and each is scored “1: None” through “4: A lot” according to the 4-point Likert Scoring System. The total score ranges from 0 to 100. The higher scores indicate worse fatigue levels or vice versa [27].

### Statistical analysis

The data was shown as means and standard deviation or number and percentages according to the data whether continuous or categorical. The normality was checked via KS-SW tests as well as skewness and kurtosis. Since three different time points were planned in this study design, repeated measures analysis of variance within factor (ANOVA) was used. In case of violated assumptions of repeated measures ANOVA, the Friedman test was used. In case of a significant value obtained according to the Friedman test, pairwise comparisons along with Bonferroni-Dunn post-hoc test adjustments were performed and reported. According to the normality assumption that was met, Pearson’s r or Spearman’s rho correlation was used to assess the correlation between outcomes. Dichotomous variables were analyzed via the Chi-square test. A priori power analysis was conducted by choosing the mean of small (0.01) and medium (0.06) partial eta squared (η_p_^2^ ~ 0.035), minimum 80% power and 0.05 alpha level according to the repeated measures ANOVA by accounting for partial eta squared [28]. The rest of the features have remained default in the program. It was shown that 54 participants needed to be included according to the output by G*Power 3.1.9.4. However, we aimed to enroll at least 60 patients by calculating the potential 10%. Although there might be various reasons for dropout, we chose to use a 10% rate for potential dropout in parallel with the literature [29]. According to the post-hoc power analysis with a total of 56 patients considering the peripheral muscle strength outcome, within 95% CI and alpha level is accepted as significant below 0.05, we achieved over 86% power (η_p_^2^: 0.034, Power: 0.8688) [30]. A *p*-value below 0.05 was accepted as statistically significant. Statistical analyses were performed with IBM SPSS 20.0 (IBM Corp, Armonk, USA).

## Results

A total of 56 patients completed all assessments from baseline to final assessment. Mean age and BMI were 51.93 (11.70) years and 28.76 (5.22) kg/m^2^, respectively. All patients were female, and they had no lymphedema during and after the completion of the study. The great majority of patients (94.6%) were right-handed. The mean duration of the chemotherapy process was 16.79 ± 4.06 weeks. The mean exposure total dose was 1411.37 ± 791.71 mg. Surgery was performed in 42 out of 56 patients. Neoadjuvant chemotherapy prior to surgery was completed in 14 patients (25%). More than half of the patients were treated with combined anthracycline and paclitaxel. Table 1 shows the baseline clinical characteristics of patients.

**Table 1:** Baseline clinical characteristics of patients

n: 56	n (%)	n (%)
	**Conservative**	**MRM**
**Type of Breast Surgery**	32 (76.2)	10 (23.8)
	**ALND**	**SLNB**
**Axillary procedure**	35 (83)	7 (17)
	**Right**	**Left**
**Side of surgery**	27 (64.2)	15 (35.8)
	**Adjuvant**	**Neoadjuvant**
**Chemotherapy**	42 (75)	14 (25)
	**TC**	**AC + PAXL**
**Chemotherapy Protocol**	24 (42.9)	32 (57.1)
	**Right**	**Left**
**Hand Dominance**	53 (94.6)	3 (5.4)
	**No**	**Yes**
**Lymphedema**	56 (100)	0 (0)

*BMI* Body mass index, *AC* Adriamycin + Cyclophosphamide, *PAXL* Paclitaxel, *TC* Taxotere + Cyclophosphamide (Docetaxel), *MRM* Modified radical mastectomy, *ALND* Axillary lymph node dissection, *SLNB* Sentinel lymph node biopsy, *Min* Minimum, *Max* Maximum

HGS was found significant among three measurements (Friedman Test χ^2^: 11.279, *p* = 0.004). Pairwise comparisons with Bonferroni adjustments revealed that HGS was found significant between T1 and T3. Peripheral muscle strength was not found significant (F (2,110) = 1.914, *p* = 0.152). The fine motor function was also found significant from baseline to final assessment (Friedman test: χ^2^: 9.893, *p* = 0.007). Pairwise comparisons with Bonferroni adjustments revealed that significant results were obtained between T1 and T2 (*p* = 0.01) as well as T2 and T3 in MMDT (*p* = 0.042). Patients receiving neoadjuvant chemotherapy showed lower time in MMDT compared to patients receiving adjuvant chemotherapy after breast cancer surgery in the baseline (67.30 vs 75.70 s, t = 2.108, *p* = 0.040). HGS, peripheral muscle strength, and MMDT values of patients are shown in Table 2.

**Table 2:** Handgrip strength, peripheral muscle strength, and Minnesota Manual Dexterity Test results of patients through three different time points

***n* = 56**	**T**_**1**_	**T**_**2**_	**T**_**3**_	**χ**^**2**^	**p**		**ES**
	**Median (IQR25-75)**	**Median (IQR25-75)**	**Median (IQR25-75)**			**Adj. p**	
**HGS (kg)**	21.92^a^	21.58	20.98^a^	11.279	**0.004**	**0.03**	0.40^d^
**MMDT (Sec)**	71.03^b^	70.04^b,c^	73.14^c^	9.893	**0.007**	**0.01**^**b**^ **0.042**^**c**^	0.35^d^
	**Mean (SD)**	**Mean (SD)**	**Mean (SD)**	**F**	**p**		**η**_**p**_^**2**^
**QF (N)**	188.89 (35.36^*^)	179.89 (34.39^*^)	182.26 (37.85)	1.914	0.155		0.034

*HGS* Handgrip strength, *QF* Quadriceps femoris, *MMDT* Minnesota Manual Dexterity Test, *T1* Time1, *T2* Time 2, *T3* Time 3, *N* Newton, *Sec* Second, *χ*^*2*^ Chi-square statistic, *η*_*p*_^*2*^ partial eta squared, *IQR* Interquartile range, *ES* Effect size, *Adj. p* adjusted *p*-value according to the Bonferroni-Dunn adjustment for pairwise comparisons. *Time 1 (T1)* Before chemotherapy, *Time 2 (T2)* Middle chemotherapy time point (approx.8 weeks later), *Time 3 (T3)* Post-chemotherapy time point (12–20 weeks later after initial assessment)

*Significant difference was obtained after pairwise comparison

^**a**^HGS was found significantly different according to the Friedman test between T1 and T3 after the posthoc Dunn test (adjusted *p*-value was found as 0.03 after Bonferroni correction)

^b^MMDT was found significantly different according to the Friedman test between T1 and T2 as well as between T2 and T3 after the Bonferroni adjustment

^c^MMDT was found significantly between T2 and T3 after Bonferroni adjustment

^d^Kendall’s W value

The cutaneous sensory tactile function was analyzed between each pre-defined reference point as applied “gram” force. Although significant results were obtained in some reference points when considering for target gram force, there were no significant differences after pairwise comparisons with Bonferroni adjustment (*p* > 0.05). It was also found that there was no significant difference was obtained from T1 to T3 in terms of the diminished protective sensation (DPS). Tables 3 and 4 show the change in each reference point in three different assessment time points, respectively. Table 3 shows the target gram force for each reference point, while Table 4 shows the numbers and percentages of patients within the corresponding label whether each reference point is within “Normal” or “DPS”.

**Table 3:** Cutaneous sensation thresholds throughout the measurement times according to the SWMT

***n* = 56**	**Reference Points**	**Median** **IQR (**^**25−75th)**^**)**			**χ**^**2**^	**p**
		**T1**	**T2**	**T3**		
**Right Hand (g)**	**1**	0.07 (0.07–0.40)	0.07 (0.07–0.40)	0.40 (0.07–0.40)	1.224	0.542
	**2**	0.07(0.07–0.23)	0.07 (0.07–0.4)	0.07 (0.07–0.4)	3.432	0.180
	**3**	0.07 (0.07–0.07)	0.07 (0.07–0.07)	0.07 (0.07–0.23)	5.733	0.057
	**4**	0.07(0.07–0.07)	0.07 (0.07–0.07)	0.07 (0.07–0.07)	2.772	0.250
	**5**	0.07(0.07–0.07)	0.07 (0.07–0.4)	0.07 (0.07–0.4)	6.400	0.041^*^
	**6**	0.07(0.07–0.07)	0.07 (0.07–0.4)	0.07 (0.07–0.4)	4.276	0.118
**Left Hand(g)**	**1**	0.07 (0.07–0.07)	0.07 (0.07–0.4)	0.07 (0.07–0.4)	11.043	0.004^*^
	**2**	0.07 (0.07–0.07)	0.07 (0.07–0.07)	0.07 (0.07–0.07)	8.522	0.014^*^
	**3**	0.07 (0.07–0.07)	0.07 (0.07–0.07)	0.07 (0.07–0.07)	7.400	0.025^*^
	**4**	0.07 (0.07–0.07)	0.07 (0.07–0.07)	0.07 (0.07–0.07)	7.538	0.023^*^
	**5**	0.07 (0.07–0.07)	0.07 (0.07–0.07)	0.07 (0.07–0.23)	3.846	0.146
	**6**	0.07 (0.07–0.07)	0.07 (0.07–0.07)	0.07 (0.07–0.07)	1.850	0.397
**Right Foot(g)**	**P1**	0.40 (0.4–0.4)	0.40 (0.07–0.40)	0.40 (0.4–0.6)	6.222	0.045^*^
	**P2**	0.40 (0.07–0.4)	0.40 (0.07–0.4)	0.40 (0.07–0.4)	6.242	0.044^*^
	**P3**	0.40 (0.4–0.4)	0.40 (0.07–0.4)	0.40 (0.4–0.6)	3.925	0.141
	**P4**	0.40 (0.4–0.4)	0.40 (0.23–0.5)	0.40 (0.4–1)	8.139	0.017^*^
	**D1**	0.40 (0.07–0.4)	0.40 (0.07–0.4)	0.40 (0.07–0.4)	0.463	0.793
	**D2**	0.07 (0.07–0.4)	0.07 (0.07–0.4)	0.40 (0.07–0.4)	4.019	0.134
**Left Foot(g)**	**P1**	0.40 (0.4–0.4)	0.40 (0.07–0.4)	0.40 (0.23–0.5)	8.060	0.018^*^
	**P2**	0.40 (0.07–0.4)	0.40 (0.07–0.4)	0.40 (0.07–0.4)	3.931	0.140
	**P3**	0.40 (0.4–0.4)	0.40 (0.07–0.4)	0.40 (0.4–0.6)	7.868	0.020^*^
	**P4**	0.40 (0.4–0.4)	0.40 (0.4–0.4)	0.40 (0.4–0.8)	7.056	0.029^*^
	**D1**	0.40 (0.07–0.4)	0.40 (0.07–0.4)	0.40 (0.07–0.4)	0.063	0.969
	**D2**	0.40 (0.07–0.4)	0.07 (0.07–0.4)	0.40 (0.07–0.4	1.046	0.593

For hand reference points, 1,2 and 3 correspond to the palmar cutaneous branch of the Ulnar nerve for hand, while reference points 4,5 and 6 correspond to the palmar cutaneous branch of Median nerve

For foot reference points, P1 and P2 correspond to the Medial plantar nerve, P3 corresponds to the Lateral plantar nerve, P4 corresponds to the Sural nerve, D1 and D2 correspond to the Deep Fibular nerve and Dorsal lateral cutaneous nerve, respectively

*χ*^*2*^ Non-parametric Friedman Test for Related Samples, *p* < 0.05, *IQR* Inter quartile range, *SWMT* Semmes Weinstein Monofilament Test, *g* Gram

^*^p values lower than 0.05 were not found significant after pairwise comparisons with the Bonferroni-Dunn test

**Table 4:** Comparison of the percentages of patients with normal or diminished protective sensation according to the SWMT in each reference point throughout the measurement times

***n* = 56**	**T1**		**T2**		**T3**			
	**n (%)**		**n (%)**		**n (%)**			
**Right Hand**	**Normal**	**DPS**	**Normal**	**DPS**	**Normal**	**DPS**	**χ**^**2**^	**p**
1	56 (100)	-	55 (98.2)	1 (1.8)	56 (100)	-	2.000	.368
2	53 (94.6)	3 (5.4)	56 (100)	-	54 (96.4)	2 (3.6)	3.500	.174
3	56 (100)	-	56 (100)	-	56 (100)	-	-	-
4	53 (94.6)	3 (5.4)	54 (96.4)	2 (3.6)	55 (98.2)	1 (1.8)	0.667	.717
5	55 (98.2)	1 (1.8)	55 (98.2)	1 (1.8)	55 (98.2)	1 (1.8)	-	-
6	54 (96.4)	2 (3.6)	55 (98.2)	1 (1.8)	56 (100)	-	2.000	.368
**Left Hand**								
1	56 (100)	-	55 (98.2)	1 (1.8)	56 (100)	-	-	-
2	55 (98.2)	1 (1.8)	56 (100)	-	55 (98.2)	1 (1.8)	1.000	.607
3	55 (98.2)	1 (1.8)	56 (100)	-	56 (100)	-	2.000	.368
4	56 (100)	-	56 (100)	-	56 (100)	-	-	-
5	55 (98.2)	1 (1.8)	56 (100)	-	56 (100)	-	2.000	.368
6	55 (98.2)	1 (1.8)	56 (100)	-	56 (100)	-	2.000	.368
**Right Foot**								
P1	56 (100)	-	56 (100)	-	56 (100)	-	-	-
P2	56 (100)	-	56 (100)	-	56 (100)	-	-	-
P3	56 (100)	-	56 (100)	-	56 (100)	-	-	-
P4	56 (100)	-	56 (100)	-	56 (100)	-	-	-
D1	54 (96.4)	2 (3.6)	55 (98.2)	1 (1.8)	55 (98.2)	1 (1.8)	1.000	.607
D2	55 (98.2)	1 (1.8)	54 (96.4)	2 (3.6)	51 (91)	5 (9)	5.200	.074
**Left foot**								
P1	56 (100)	-	56 (100)	-	56 (100)	-	-	-
P2	56 (100)	-	56 (100)	-	56 (100)	-	-	-
P3	56 (100)	-	56 (100)	-	56 (100)	-	-	-
P4	56 (100)	-	56 (100)	-	55 (98.2)	1 (1.8)	2.000	.368
D1	56 (100)	-	55 (98.2)	1 (1.8)	53 (94.6)	3 (5.4)	3.500	.174
D2	55 (98.2)	1 (1.8)	55 (98.2)	1 (1.8)	52 (92.8)	4 (7.2)	3.600	.165

*Time 1 (T1)* Before chemotherapy, *Time 2 (T2)* Middle chemotherapy time point (approx.8 weeks later), *Time 3 (T3)* Post-chemotherapy time point (12–20 weeks later after initial assessment)., *DPS* Diminished Protective Sensation, *χ*^*2*^ Cochran Q-test, *p* < 0.05

Fatigue significantly increased between T1 and T3 (19.44 vs 27.77, z: -2.347, *p* = 0.019, ES: -0.31). Mean HGS, peripheral muscle strength, MMDT, and total chemotherapy duration were not found to correlate with fatigue both in T1 and T3. (*p* > 0.05). Yet, when comparing the mean differences (T1-T3) between those outcomes, only fatigue and HGS were found significantly correlated (*r* = 0.385, *p* = 0.003).

Chemotherapy duration tended to increase as neoadjuvant according to the point-biserial correlation (*r* = 0.504, *p* < 0.001) while the duration of chemotherapy seemed to be lower in patients with higher age (*r* = -0.391, *p* = 0.003). Age was found to correlate significantly with BMI (*r* = 0.385, *p* = 0.03) and with mean HGS in all measurement points (*r* = -0.470, -0.443, and -0.422 *p* < 0.001 through T1 and T3, respectively). Age was also found significantly correlated with PMS in T3 (*r* = -0.297, *p* = 0.026). Fine motor function was found significantly correlated with age in all three measurement points (*r* = 0.596, 0.485, and 0.489 *p* < 0.001 from T1 through T3, respectively). MMDT was significantly correlated with mean HGS in both the baseline (T1) (*r* = -0.475, *p* < 0.001) and final measurement (T3) (*r* = -0.379, *p* = 0.004).

## Discussion

This study showed significant fair to moderate acute effects of systemic chemotherapy on sensorimotor functions of breast cancer patients as decreased handgrip strength, deteriorated fine motor function, increased burden of fatigue, and diminished cutaneous sensation.

Chemotherapy agents especially anthracyclines which are frequently used in breast cancer are known as an important factor for muscle loss and diminished activity tolerance [31, 32]. In parallel with this, general muscle strength which was assessed with HGS was found significant from baseline through the end of chemotherapy in our study. This result was anticipated because the cumulative effects of chemotherapy possibly played a key role in the reduced general muscle strength. [33]. Not surprisingly, it was also reported that decreased handgrip strength is evident after breast cancer surgery [34, 35]. In parallel with this, the great majority of our sample (75%) had undergone breast cancer surgery along with ALND, and therefore, decreased muscle strength might have been related to this situation due to ongoing impairments caused by ALND [36]. Kootstra et al. [37] reported that women who had ALND had worse shoulder strength-related function than women who had SLNB in a seven-year follow-up study. However, peripheral muscle strength was also decreased midway through the systemic chemotherapy process, however, this decrease did not reach statistical significance. Vardar-Yağlı et al. [38] reported that peripheral muscle strength was found significantly associated with comorbidity index as well as physical activity and depression level among breast cancer survivors. Yet, the authors also reported no significant relationship between peripheral muscle strength and functional capacity. In our study, we also did not find a significant relationship between peripheral muscle strength and fine motor function.

Older cancer survivors frequently experience more bothersome symptoms compared to younger cancer survivors. Hoppe et al. [39] reported even one dose of chemotherapy application results in functional decline at the rate of 17% among elderly breast cancer survivors. Owusu et al. [40] reported that a one kg decrease in handgrip strength causes functional limitation among older breast cancer survivors at the rate of 18%. Ying et al. [41] reported a 2.6 m decreased total walked distance as per unit increase in age. In parallel, we also found significant relationships between age, fine motor function, HGS, and peripheral muscle strength. These significant findings of our study highlight age as a major factor that should be kept in mind not only short term but also in the long term of survival.

SWMT primarily tests Aβ fibers, thereby in our study, we chose to use SWMT to detect potential CIPN-related sensory changes due to the high rate of CIPN that may be seen with the taxane-based medication paclitaxel [42]. Although significant results were obtained in some reference points, those were not found significant in pairwise comparisons. Since we focused on especially in potential acute effects by doing the final assessment after completion of chemotherapy within 7–10 days, cumulative effects of chemotherapy-related sensory disturbances might not have manifested. Griffith et al. [24] also reported no significant difference in tactile detection threshold in patients with cancer with or without CIPN. Da Silva Simão et al. [7] reported a significant difference in SWMT in patients who underwent chemotherapy, though this result can be disputable because the authors included patients who had already taken at least three dosages of taxanes at the baseline in their study.

CIPN is reported to be as having prominent symptoms that usually occur in the upper extremities later than in lower the extremities [17]. The plantar surface of the foot is reported more susceptible to CIPN-related sensorial deterioration according to Da Silva Simão’s study [7], yet we did not find any significant difference in the plantar surface. In our study, we did not use any patient-reported outcome measure related to CIPN and thus we could not infer the comparative efficacy of SWMT. Nonetheless, studies reported both patient-reported outcomes such as the Chemotherapy-Induced Neurotoxicity Questionnaire (CINQ) and SWMT are capable of tracking changes related to CIPN [19]. Since we aimed to assess pre-clinical CIPN, we chose to use SWMT as an objective outcome. The second factor that led to the selection of SWMT was the fact that paclitaxel, another common treatment in our sample, is known to have the highest prevalence of CIPN, and related issues are typically sensory rather than motor and autonomic [43]. Studies also stated that patient-reported outcomes can detect the effects of CIPN on daily living and quality of life instead of detecting early signs of CIPN. SWMT has also been stated as an effective tool to track early changes compared to patient-reported outcomes [44].

Cancer-related fatigue (CRF) is a major problem among patients with cancer actively undergoing chemotherapy and/or cancer survivors. CRF is multifactorial, yet chemotherapy agents cause myotoxic effects thereby decreased muscle mass cannot generate efficient strength even for daily life activities [45]. Kilgour et al. [46] stated that CRF is directly linked to muscle mass and strength in patients with advanced-stage cancer. It was reported that not only peripheral but also centrally originated fatigue can cause decreased strength, however, mitigating fatigue with increased strength is debatable [47]. Yet, it should be noted that exercise plays a crucial role in fatigue. In a meta-analysis that included 3418 patients, it was reported that a decrease in fatigue and improved physical functioning was achieved via exercise intervention [48]. Similar studies also support that exercise has a remarkable effect on improved cancer-related fatigue [49, 50]. Most recently, Hucteau et al. [51] reported that the diminished exercise capacity was the result of central fatigue in patients with breast cancer who underwent chemotherapy. Koevoets et al. [52] also reported that exercise intervention improved self-reported cognitive function. In our study, we did not find any significant relationships among fatigue, HGS, PMS as well and MMDT in both baseline and final measurements. However, fatigue was found significantly increased from T1 through T3. This result was not surprising since the cumulative effect of systemic treatment naturally affected this result. Though EORTC-FA12 is reported as an optimal instrument for CRF [53], most items of EORTC-FA12 are not directly linked to fatigue which originates from the musculoskeletal system and therefore EORTC-FA12 might not be the optimal choice in some patients groups. Recall bias or unstable perception of fatigue during chemotherapy might have influenced these insignificant results. Nevertheless, mean differences in fatigue and HGS (ΔT1-T3) were found significantly correlated which can be interpreted as the greater decrease in HGS affects the perception of fatigue. However, the literature supports our finding that no or very weak correlations were reported between handgrip strength and fatigue among cancer patients [53, 54].

Diminished hand function might be a detrimental factor for optimal functionality. MMDT has been widely used in patients with different kinds of areas such as neurological deficits, hand surgery, hand preference, industrial work performance, rehabilitation, etc. [26]. To the best of our knowledge, MMDT has not been used in cancer patients at risk for CIPN. We hypothesized that potential CIPN can affect both hands, and pre-clinical CIPN may affect the performance level of MMDT. We found significant deteriorations in terms of times between T1 and T2 as well as T2 and T3 in fine motor function. In addition, patients who underwent neoadjuvant chemotherapy showed significantly better results compared to patients who underwent adjuvant chemotherapy. This result can be expectable due to the relatively short duration between surgery and adjuvant treatment period; thus, these patients can be thought of as in still recovery process regarding pain or stiffness of the surgical side and/or axilla due to surgery. Besides, we found that baseline HGS was significantly correlated with MMDT at a moderate level. This finding can be explained that when performing MMDT, due to the time-dependent nature of this test it is also needed optimal upper extremity strength.

Since we aimed to assess changes in sensorimotor functions before, during, and after chemotherapy, we did not include any CIPN-related patient-reported outcome, and therefore we cannot conclude whether patients suffer from acute CIPN or not at the end of this study. This might be thought of as a limitation of this study since we might have missed the autonomic symptoms of potential CIPN such as tingling, numbness, etc. Recall bias and/or arbitrarily filling out patient-reported outcomes (EORTC-FA12) might be thought of as a limitation. In addition, we did not assess fatigue in the middle of the chemotherapy process, therefore it can also be counted as a limitation. However, we might have failed to show the cumulative effect of chemotherapy especially for patients who underwent four cycles of Docetaxel treatment in 21-day intervals if we had assessed fatigue after two cycles of Docetaxel treatment. In addition, we also wanted to eliminate the recall bias. Yet, it was reported that fatigue acts in a highly variable pattern in the process of chemotherapy which shows itself highest following week after chemotherapy infusion, then decline is observed through the next infusion [55]. Potential sampling biases can also be counted as a limitation. We only enrolled patients in one outpatient oncology service, therefore it's debatable if our findings are generally applicable. A rather small sample size can also be considered a constraint, despite the fact that we were able to reach the requisite power above 80%. However, to our knowledge, this is the first study into how chemotherapy might affect fine motor function, which was measured using a reliable and objective method.

Due to the chronic nature of CIPN, it must be extensively evaluated in oncology settings both during the active treatment phase and throughout survivorship. During the active treatment period, rehabilitation in cancer settings may offer crucial insight to spot early CIPN alterations. Thus, the quality of cancer care, as well as the prevention of the deteriorating effects of CIPN, might be achieved. In addition, SWMT and MMDT are shown they can be used safely, and they can be another option to detect potential pre-clinical CIPN-related deteriorations in patients with breast cancer who are at risk for CIPN. Long-term evaluation of sensorial and fine motor function and follow-up with these tests may be within the scope of further studies.

## Data Availability

The data can be available from the corresponding author upon reasonable request and with permission of Bakircay University Ethical Board of Clinical Studies.

## Abbreviations

ALND: Axillary lymph node dissection
ANOVA: Analysis of variance
BMI: Body mass index
CI: Confidence interval
CIPN: Chemotherapy induced peripheral neuropathy
CRF: Cancer-related fatigue
CT: Chemotherapy
EORTC-QLQ-FA12: European Organization for Research and Treatment of Cancer Quality of Life Module Cancer Related Fatigue
HGS: Handgrip strength
MMDT: Minnesota Manual Dexterity Test
N: Newton
η_p_^2^: Partial eta squared
χ^2^: Chi-square
PMS: Peripheral muscle strength
SWMT: Semmes-Weinstein Monofilament Test
QF: Quadriceps femoris
T1: Time 1
T2: Time 2
T3: Time 3
